# Comparing the history of signalling pathway research using the research publication record of representative genes.

**DOI:** 10.17912/micropub.biology.000998

**Published:** 2023-10-27

**Authors:** Helen Attrill

**Affiliations:** 1 Department of Physiology, Development and Neuroscience, University of Cambridge, Cambridge, England, United Kingdom

## Abstract

The development and application of genetic techniques in the fruit fly *Drosophila melanogaster* underlies some major advances in the understanding of metazoan development and biology. To examine whether the publication record for signalling pathway genes can indicate which factors have shaped pathway research, the publication history of selected genes is used to compare differences in research output over time. This is used to discuss how research trends may be shaped by a variety of factors such as advances in technology, ease of study and importance to human health.

**Figure 1. The publication history of signalling pathway genes.**
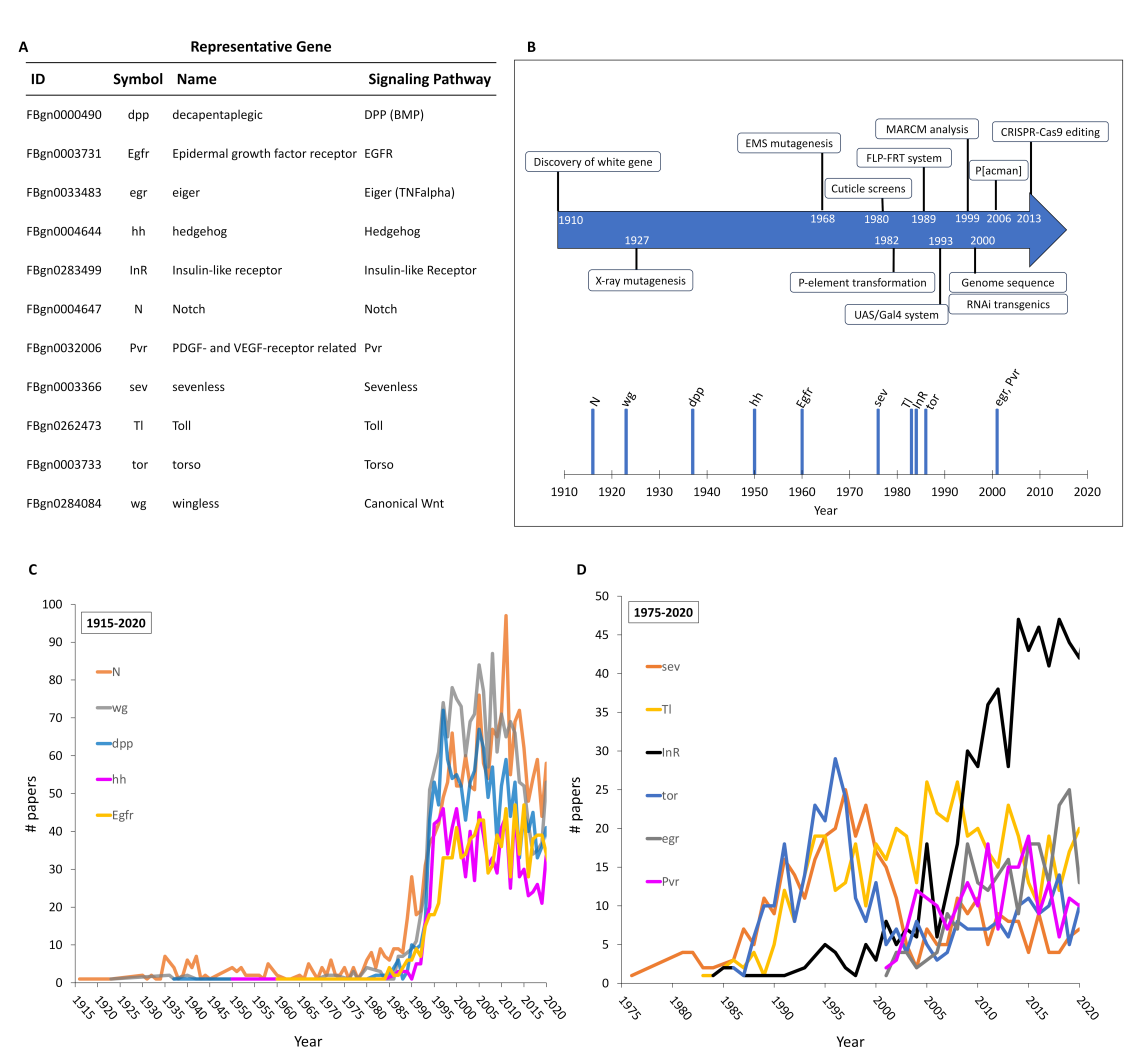
(A) A table of the genes selected as representative signalling pathway genes. (B) Selected advances in Drosophila research technology are compared (arrow, top; adapted from Bellen et al., 2010, with additional information from Mohr, 2018) with a timeline of the discovery of representative pathway genes (bottom). Graphs (C, D) plotting the number of research papers associated with one selected pathway member gene against publication year. The results are displayed in two different plots, broadly grouped based on year of first publication. In (C), the publication plots for *N* , *wg* , *dpp* , *hh* and *Egfr* , which were first published on between 1916 and 1960, are shown; in (D) the plots for the RTK pathways *tor* , *sev* and *InR* , first published mid-1970s-mid-1980s; *Tl* (1983) and *Pvr* , *egr* (2001), are shown. Note the different axes for graphs (C) and (D).

## Description

*Drosophila* has been central to the discovery and elucidation of many key signalling pathways (Bellen, et al. 2010) . As such, pathway research in *Drosophila* may provide a good starting point to compare and examine research trends of genes with a common functional role but with different histories. To do this, a defining/prototypic gene was selected as a representative of eleven different pathways ( Fig. 1A ), for example, the *Notch* ( *N* ) gene for the Notch signalling pathway and *wg* for the canonical Wnt signalling pathway. The research output over time was compared by plotting the number of research papers published/year for each gene by using gene publication data in FlyBase (flybase.org, Gramates et al., 2022). The most intensely studied genes, which were all first described between 1916 and 1960 ( Fig. 1B, bottom panel ): *N* (Morgan and Bridges, 1916) , *wg* (Mann, 1923) , *dpp* (Novitski and Rifenburgh, 1937) , *hh* (Ives, 1950) and *Egfr* (Grell 1960) , display a similar trajectory of study ( Fig. 1C ). After the initial description there was a span of low, periodic output. This began to gradually rise early in the 1980s with a steeper rise towards end of this decade, which continued through the mid-1990s to 2000, and then plateaued at a relatively high rate, with a small decline post-2015. The study of these important and long-studied genes reflects some of the major advances in Drosophila genetics ( Fig. 1B, top panel ), progressing from ethyl methanesulfonate mutagenesis developed in the late 1960s (Lewis and Bacher, 1968) , the application of forward genetic screens and development of new techniques drove a rise in the 1980s, for example the genetic screens for mutations affecting cuticle patterning revealing some of the major players in segment polarity (Nüsslein-Volhard and Wieschaus, 1980), the generation of insertion alleles through P-element transformation (Rubin and Spradling, 1982) , efficient site-directed mitotic recombination via the FLP-FRT system (Chou and Perrimon, 1992; Xu and Rubin, 1993) and the Gal4/UAS system for controlled ectopic expression (Brand and Perrimon, 1993) . With the release of the complete fly genome sequence in 2000 (Adams et al., 2000) , the post-genomics era saw the employment of reverse genetics tools, such as RNAi (Kennerdell and Carthew, 2000; Boutros et al., 2004) and CRISPR/Cas9, for targeted gene studies (Ren et al., 2013; Bassett et al., 2013; Gratz et al., 2013) and the detailed molecular dissection of these signalling pathways.

The genes *sev* and *tor* display a very different publication profile. They were first described in the mid-1970s (Harris et al., 1976) and 80s (Schupbach and Wieschaus, 1986) , respectively ( Fig. 1B, bottom panel ). From discovery, there was a steady increase in publication with a clear peak in the mid- to late 1990s followed by a pronounced decrease around the turn of the century ( Fig. 1D ). The research profile of these genes may be due to two common factors. First, the intense period of study may reflect that these pathways were used as the major workhorses to genetically dissect canonical receptor tyrosine kinase (RTK)-Ras/Raf/MAP kinase signalling, owing to their easily scorable phenotypes and very precise localised action. Second, the lack of sustained interest in these pathways may be in part because they are more phylogenetically restricted than other RTK pathways. The Torso terminal signal pathway is not used beyond a limited set of arthropods (Duncan et al., 2013) . A similar situation is true for the Sevenless signalling pathway, despite the existence of a vertebrate ortholog of *sev* - the orphan receptor tyrosine kinase ROS1 (human ortholog HGNC:10261); the gene encoding the ligand of sev, *boss* , is not found outside of a limited set of insect species (Bao and Friedrich, 2008) . Thus, the lack of functional conservation with humans may have contributed to the waning of interest.

In common with *tor* and *sev* , publications for *Tl* , *egr* and *Pvr* (first described by Campos-Ortega, 1983, Aravind et al., 2001 and Heino et al., 2001, respectively ( Fig. 1B, bottom panel )) show a general trend for an increase in papers following the first description of the gene ( Fig. 1D ). However, rather than a steep decline in publications, the research output tends to plateau, suggesting continued research interest. In common with the more intensely studied genes shown in Fig. 1C, the research output on the *InR* gene shows a period of low research intensity followed by a sustained rise to high levels ( Fig. 1D ). However, unlike these early-discovered genes, *InR* was described much later (Mohler and Pardue, 1984) , when genetic tools such as FLP-FRT and UAS-Gal4 were more readily available. Compared with the research publication profile of the *tor* and *sev* , both also encoding RTKs and discovered around a similar time (mid-70s to mid-80s), there is a profound difference. As discussed above, publication rate for *tor* and *sev* rose immediately after discovery, peaking in the 1990s and waning by the early 2000s. However, *InR* had very little study until the mid-2000s – pointing to other influences beyond technological limitations. Prior to that time, *Drosophila* was not regarded as a good model for human insulin signalling and therefore was largely ignored by the field (Graham and Pick, 2017) . However, that view changed, and *Drosophila* gained popularity as a model for the role of the insulin pathway in obesity and ageing - the *InR* research publication rate rose rapidly between 2005 and 2015, reaching similar levels to the more highly studied pathway genes such as *N* and *wg* .

In conclusion, comparison of the publication profile of Drosophila pathway genes shows that for some the research output follow common trajectories, while for others, very different trends are evident. Genes described early in the 20 ^th^ century, such as *N* and *wg* , were discovered because of the visible phenotypic effects of their mutants. The impact of molecular genetics on study of these genes and their cognate pathways is reflected in the steep rise in publication output during the 1980s. Their continued prominence in research reflects the importance of these signalling pathways in development and human health. For other genes described later, technological limitations may have a smaller influence on study and other factors, such as their popularity as tractable model systems and/or relevance to human health, may be more dominant factors in shaping their study.

## Methods

Genes were selected as ‘representative’ of a pathway such that research on the gene and research of its cognate pathway would be regarded as intimately associated. In this way the number of papers may serve as a proxy measure (but by no means an exact measure) for the pathway’s research and the discovery of the representative gene would be considered by most to coincide with the beginning of research on that pathway. Several criteria were used to select genes: (1) the gene encodes a ligand or receptor (other classes of gene such as transcription factor or kinase are often shared by multiple processes and would therefore not be suitable); (2) the gene must have no or extremely limited functions beyond its associated signalling pathway; (3) ideally, the gene should have no functional redundancy in the pathway e.g. Egfr is the sole receptor of the EFGR pathway. If this is not possible, the dominant form employed by the pathway should be selected e.g., *wg* for the canonical Wnt signalling pathway. These choices were aided by examining number of associated pathway papers from the FlyBase Signalling Pathway resource (http://flybase.org/lists/FBgg/pathways (Attrill et al., 2023) ).

For assessing the number of research papers published/year for each representative gene, research papers were exported from the reference section of the corresponding gene report in FlyBase (release FB2022_06) to a HitList. The year of publication for each paper was obtained using FlyBase Batch Download and the number of publications/year collated using the PivotTable function in Microsoft Excel.

Note: For this work, data from FlyBase was deemed more reliable for finding a complete list of research publications on Drosophila genes over other available research paper resources. First, the Drosophila genes studied in research papers have been associated with papers by expert curators. Second, FlyBase has assembled publications that have not been indexed by PubMed and/or do not have a DOI such as the Drosophila Information Service ( https://www.ou.edu/journals/dis/) . This was particularly important for finding early reports describing mutant flies. For more detailed information on the FlyBase bibliography see Marygold et al., 2012.
